# Marginal and Internal Fit of Ceramic Restorations Fabricated Using Digital Scanning and Conventional Impressions: A Clinical Study

**DOI:** 10.3390/jcm9124035

**Published:** 2020-12-14

**Authors:** Jeong-Hyeon Lee, Keunbada Son, Kyu-Bok Lee

**Affiliations:** 1 Department of Prosthodontics, School of Dentistry, Kyungpook National University, 2177 Dalgubeol-daero, Jung-gu, Daegu 41940, Korea; prossn@naver.com; 2 Advanced Dental Device Development Institute (A3DI), Kyungpook National University, 2177 Dalgubeol-daero, Jung-gu, Daegu 41940, Korea; sonkeunbada@gmail.com; 3 Department of Dental Science, Graduate School, Kyungpook National University, 2177 Dalgubeol-daero, Jung-gu, Daegu 41940, Korea

**Keywords:** marginal and internal fit, intraoral scanner, conventional method, ceramic crown, digital workflow

## Abstract

This clinical study was designed with the aim of fabricating four ceramic crowns using the conventional method and digital methods with three different intraoral scanners and evaluate the marginal and internal fit as well as clinician satisfaction. We enrolled 20 subjects who required ceramic crowns in the upper or lower molar or the premolar. Impressions were obtained using digital scans, with conventional impressions (polyvinyl siloxane and desktop scanner) and three different intraoral scanners (EZIS PO, i500, and CS3600). Four lithium disilicate glass-ceramic crowns were fabricated for each patient. In the oral cavity, the proximal and occlusal adjustments were performed, and the marginal fit and internal fit were evaluated using the silicone replica technique. The clinician satisfaction score of the four crowns was evaluated as per the evaluations of the proximal and occlusal contacts made during the adjustment process and the marginal and internal fit. For statistical analysis, the differences among the groups were analyzed with one-way analysis of variance and Tukey HSD test as a post-test; Pearson correlation analysis was used for analyzing the correlations (α = 0.05). There was a significant difference in the marginal and internal fit of the ceramic crowns fabricated using three intraoral scanner types and one desktop scanner type (*p* < 0.001); there was a significant difference in the clinician satisfaction scores (*p* = 0.04). The clinician satisfaction score and marginal fit were significantly correlated (absolute marginal discrepancy and marginal gap) (*p* < 0.05). An impression technique should be considered for fabricating a ceramic crown with excellent goodness-of-fit. Further, higher clinician satisfaction could be obtained by reproducing the excellent goodness-of-fit using the intraoral scanning method as compared to the conventional method.

## 1. Introduction

In the processes of fabricating and restoring prostheses, it is crucial to take impressions accurately [1,2,3]. The use of conventional impression material for taking impressions causes the patient discomfort, such as gagging; further, there may be various problems, such as the possibility of deformation of the impression material and contamination by the saliva and blood in the oral cavity [3,4]. In contrast, the use of an intraoral canner for taking a digital impression is a method that obtains impressions via direct scanning [5,6]. Moreover, it is possible to correct it and check the bite, looking at the three-dimensional (3D) virtual cast displayed in real time on the monitor [7]. In the conventional method that uses impression material, dental stone, investing material, and alloy, etc., there are differences in the expansion and contraction rate of each material; therefore, the goodness-of-fit of the prostheses may differ, depending on the proficiency of the dental technician [8]. However, prosthesis fabrication using an intraoral scanner offers the advantage in that the work process can be standardized [9]. Furthermore, digital impression taking is unlikely to cause the deformation of the impression material, and the additional scan and work are easy [9]. In addition, compared to conventional impression taking, this method involves a lower cost and less time; thus, this method tends to be used increasingly [10].

An intraoral scanner is an essential tool in chairside computer-aided design and computer-aided manufacturing (CAD/CAM) system because it allows the acquisition of a virtual cast directly from the patient’s oral cavity without the need for any additional work process [11,12,13,14,15]. In the dental clinic, where quick fabrication is necessary, lithium disilicate ceramic material is preferred because it takes less milling time and less crystallization time [16]. The lithium disilicate ceramic crown is superior to a zirconia crown in terms of better aesthetics, faster fabrication, and easier post-processing process [17,18].

The accuracy of the impression obtained from the patient’s oral cavity is important for the fabrication of a well-fitting prosthesis [13,14,15,16]. The deformation of the impression may lead to poor marginal and internal fit of the prostheses along with inaccurate working cast fabrication [13,14]. This may cause issues, such as plaque deposits in the oral cavity, secondary caries, cement dissolution, and periodontal disease [13]. Furthermore, the poor internal fit may cause loss of the retention force of the prostheses and lower fracture resistance [14,16]. In general, it is judged that the clinically allowable marginal fit of the fixed prostheses is 100–120 µm [17,18,19,20]. Previous studies fabricated zirconia coping, using an intraoral scanner, and reported about 100 µm as the marginal fit [21,22]. Many earlier trials have shown various results of the marginal fit [23,24,25,26]; however, few studies have studied the marginal and internal fit of the crowns fabricated using various intraoral scanners.

Several studies have compared the conventional method to the digital method [27,28,29,30,31]. However, the results were different and inconsistent [29,30,31]. Some studies have demonstrated superior accuracy of the conventional method [23,24,25], and some have shown better results using the digital method [27,28]. However, most previous studies were in vitro trials that performed extraoral evaluations, and various conditions that might be reflected in the oral cavity (obstacles, such as saliva, limited scan space, tongue, and cheek) have not been reflected [23,24,25]. Thus, in vivo studies conducted directly in the oral cavity with the conventional method of impression taking and intraoral scanning are continuously needed. In addition, most previous studies have compared the marginal and internal fit of the crowns fabricated using the conventional and digital method. There is insufficient research on the comparison of the ceramic crowns fabricated with different intraoral scanners [23,24,25].

In the present clinical study, we fabricated four ceramic crowns for each patient using the conventional method and the digital method using three different intraoral scanners and compare the marginal and internal fit of the ceramic crowns with respect to clinician satisfaction. The first null hypothesis is that there would be no differences in the marginal and internal fit of the prostheses fabricated using the conventional and digital methods. The second null hypothesis is that the marginal and internal fit of the ceramic crowns and clinician satisfaction were not correlated.

## 2. Materials and Methods

The present clinical test was conducted after obtaining approval from the IRB of the Kyungpook National University Dental Hospital (Approval Number: KNUDH-2019-02-02-02). The present clinical study was conducted from April 2019 to April 2020. Twenty subjects (10 women and 10 men) who required a ceramic crown on the upper or lower molar or premolar were enrolled. Of the participants, those with poor oral hygiene or more than one crown; those with parafunctional activities, such as bruxism, clenching, and grinding; those with acute or chronic temporomandibular joint dysfunction sensation or mental abnormalities; and those with serious medical conditions; as well as those who were a pregnant or breastfeeding were excluded. Based on the pilot experiment, training was provided for all the processes involved in the fabrication of ceramic crowns; using power software (G*Power version 3.1.9.2; Heinrich-Heine-Universität Düsseldorf, Düsseldorf, Germany), 20 participants were selected for the analyses (actual power = 96.1%; power = 95%; α = 0.05).

All the subjects were trained for intraoral scanning, crown design using CAD software, and all digital workflows, including the CAM process in advance. All the intraoral processes were prepared by one skilled dentist. The dentist was blinded to the information about the type of crown. Abutment teeth were ground as per the standard crown treatment guidelines [32]. All the abutment teeth were ground, using diamond rotary cutting instruments (852.FG.010; Jota AG, Rüthi, SG, Switzerland) to have 0.5-mm supragingival finish line, 2-mm occlusal reduction, and 6-degree convergence angle; the line range was rounded.

**Figure 1:**
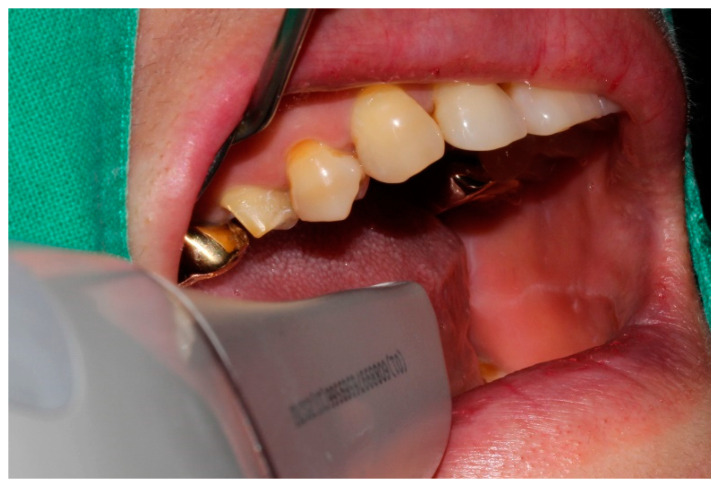
Intraoral scanning.

Impressions were obtained, using a conventional impression (desktop scanner) and three intraoral scanner types for each patient (Figure 1). All the scanners used in the present clinical study were calibrated immediately before performing scanning, and scanning was done under uniform conditions of ambient light and surface condition of the dried tooth by a single skilled operator. The same skilled dentist (J.-H.L.) conducted all the clinical tests, and four ceramic crowns were fabricated for each patient.

Group 1: Conventional impression (polyvinyl siloxane (PVS) (Aquasil Ultra; Dentsply Sirona, Bensheim, Germany) (PVS group)
Group 2: Intraoral scanner (EZIS PO; DDS, Seoul, South Korea) (EZIS PO group)
Group 3: Intraoral scanner (i500; MEDIT, Seoul, South Korea) (i500 group)
Group 4: Intraoral scanner (CS3600; Carestream Dental, Atlanta USA) (CS3600 group)

For the PVS Group, an impression was taken, using PVS impression and a double-arch tray (Dual Arch Impression Tray; 3M, MN, USA) in the oral cavity. For the material of the obtained impression, a working cast was fabricated, using Type IV dental stone (FUGIROCK; GC, Leuven, Belgium). The fabricated working cast was scanned with a desktop scanner (E1; 3Shape, Copenhagen, Denmark) and converted to an STL file. All the processes of the working cast fabrication, desktop scanning, and ceramic crown fabrication were performed by a skilled dental technician. For Intraoral Scan Groups, three intraoral scanner types were used, including EZIS PO, i500, and CS3600. All the digital scans were performed as per the manufacturers’ instructions. All the intraoral scanning processes were performed by a skilled dentist (J.-H.L.).

**Figure 2:**
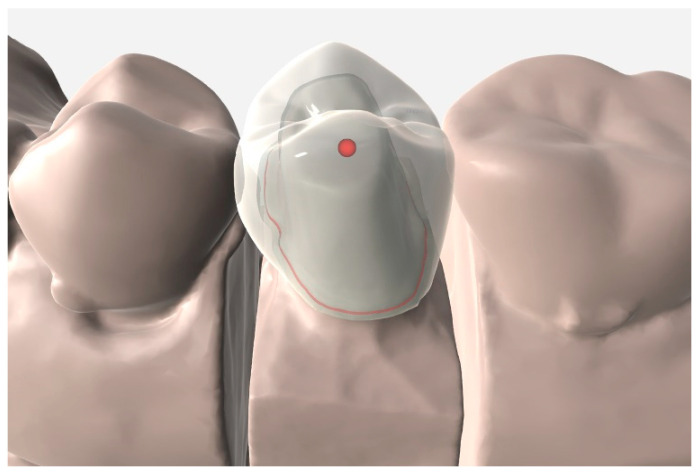
Computer-aided design of a crown.

In the scan file obtained from each group, the cement space was set at 80 µm in the dental CAD software (EZIS VR; DDS, Seoul, Korea), and the crown was designed for the anatomical shape (Figure 2). After the design preparation was complete, crowns were fabricated, using four-axis milling equipment (EZIS HM; DDS, Seoul, Korea). For the crown material, lithium disilicate glass-ceramic block (IPS e.max CAD; Ivoclar Vivadent AG, Schaan, Liechtenstein) was used. The milled ceramic crown was cleaned using the method recommended by the manufacturer, and it was crystallized and finished. Four ceramic crowns were fabricated for each patient, and 80 ceramic crowns were fabricated for 20 patients.

**Figure 3:**
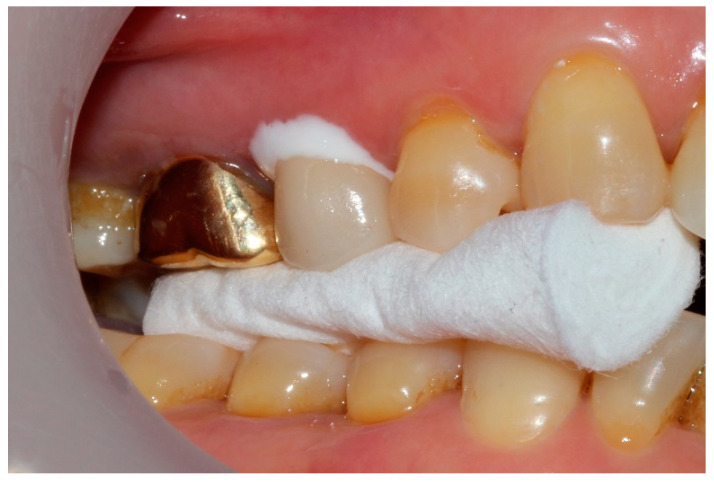
Taking silicone film for checking the fit.

Four crowns were tried for each patient intraorally and adjusted to enable optimum proximal and occlusal contacts. The marginal and internal fit for the adjustment and cementation in the oral cavity was subjected to clinical evaluation. The marginal fit was checked for appropriateness by probing with a dental explorer (5 XTS™ EXPLORER; Hu-Friedy, Chicago, IL, USA), and the internal fit was checked with silicone paste (Fit Checker; GC, Tokyo, Japan). After trying that in by putting the silicone paste, it was hardened under the patient’s bite force (Figure 3), and the transparent region of the silicon was marked on the intaglio surface of the crown, using graphite and adjusted with a diamond rotary cutting instrument. Finally, the occlusal contacts of the ceramic crown were adjusted. Using articulating paper (AccuFilm II; Parkell, Inc., Farmingdale, NY, USA), the regions of earlier contacts or interference were checked during the centric occlusion and eccentric occlusion and carefully removed, using diamond rotary cutting instruments. Using shim stock foil, proximal, and occlusal contacts were checked, and the final grinding was performed as per the recommendation of the manufacturer of the lithium disilicate glass-ceramic.

**Figure 4:**
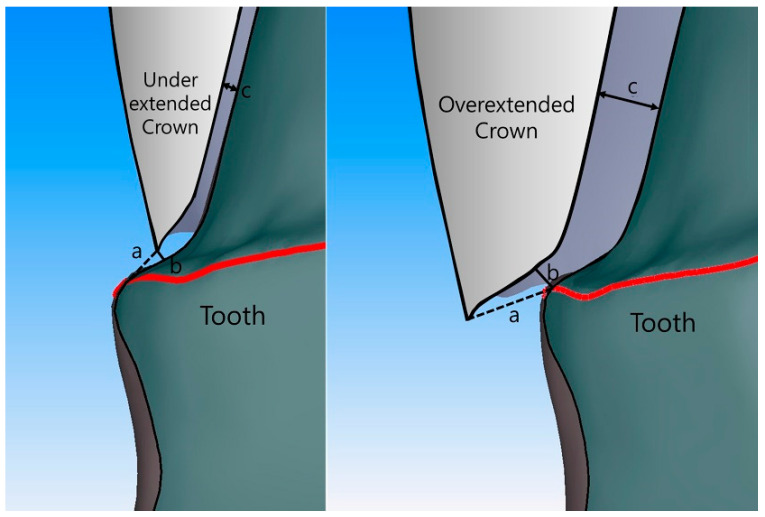
Schematic showing measurement positions for marginal and internal fit. a, Absolute marginal discrepancy. b, Marginal gap. c, Internal gap.

For each patient, the marginal and internal fit of four ceramic crowns was evaluated, using the silicone replica technique. After all the adjustments were made, silicone indicator paste (Fit Checker; GC, Tokyo, Japan) was injected into the intaglio surface of each crown, and the position of the crown was maintained at the patient’s bite force till the end of the silicon polymerization process. After the hardening of the silicone indicator paste, the ceramic crown was removed, and a light-body PVS impression was (Aquasil Ultra; Dentsply Sirona, Bensheim, Germany) was injected in the intaglio surface of the crown and hardened for five minutes to support the thin silicon layer. The silicon in which the space between the ceramic crown and the abutment tooth was duplicated was cut from the crown in the medial-mesiodistal and buccolingual directions, and the gap was evaluated at 60× magnification with an industrial video microscope system (IMS 1080P; SOMETECH, Seoul, Korea). With respect to the position of measurement, marginal fit (absolute marginal discrepancy and marginal gap) and internal fit (chamfer, axial, angle, and occlusal gap) were measured (Figure 4). In the internal fit, the chamfer gap was evaluated at the central point of the chamfer region, and the angle gap was assessed at the central point of the angle region. The axial gap was evaluated at the central point between the chamfer gap and the angle gap, and the occlusal gap was measured at the center of the occlusal region and the central point of the axial gap.

One prosthetic dentist evaluated the quality of the four crowns for each patient based on the clinician satisfaction score. The order of the four crowns was set based on the evaluations of the proximal and occlusal contacts performed in the adjustment process and the marginal and internal fit. The crown showing the best quality was assigned four points and that with the lowest quality was scored one point. The best ceramic crown was chosen and cemented as per the standard prosthetic protocol.

All the data were analyzed using SPSS statistical software (IBM, Armonk, NY, USA). First, the normality of the data was investigated using Shapiro–Wilk test. The data were normal, and the equality of dispersion was evaluated using the Levene test. As per the result, the differences among the groups were analyzed using One-way ANOVA and Tukey HSD test as a post-test (α = 0.05).

Moreover, in order to analyze the correlations between the marginal and internal fit and the clinician satisfaction score, Pearson correlation analysis was used. The correlations were divided as per the size of the Pearson correlation coefficient (PCC), as reported previously [33]. The results of the correlation analysis among the variables were explained through the following criteria by the previous studies [14,16,33]: perfect (PCC = +1 or −1), strong (PCC = +0.7–+0.9 or −0.7–−0.9), moderate (PCC = +0.4–+0.6 or −0.4–−0.6), and weak (PCC = +0.1–+0.3 or −0.1–−0.3) (α = 0.05).

## 3. Results

**Figure 5:**
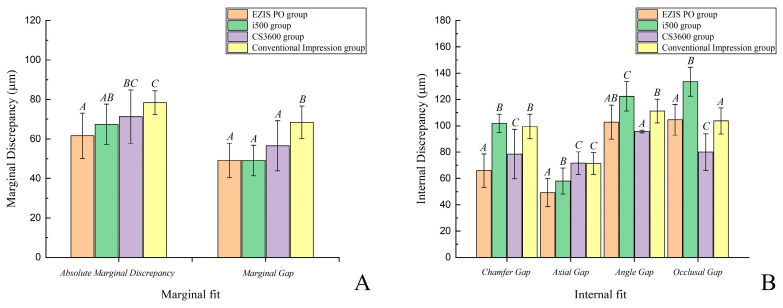
Comparison of the marginal and internal fit of ceramic restorations. (**A**) Marginal fit. (**B**) Internal fit. Same uppercase letters (A, B, C) are not significantly different (*p* > 0.05).

There were significant differences in the marginal and internal fit of the ceramic crowns fabricated using three intraoral scanner types and one desktop scanner type (*p* < 0.001; Figure 5; Table 1). There was no significant difference in the marginal gap as per the three intraoral scanner types (*p* > 0.05; Figure 5; Table 1). There was a higher value for the gap of the marginal fit (absolute marginal discrepancy and marginal gap) in the desktop scanner as compared to that in the three intraoral scanner types (*p* < 0.001; Figure 5; Table 1). There was a significant difference in the internal fit based on the three intraoral scanner types and the desktop scanner (*p* < 0.001); however, no special tendency was observed among the groups (Figure 5; Table 1).

**Figure 6:**
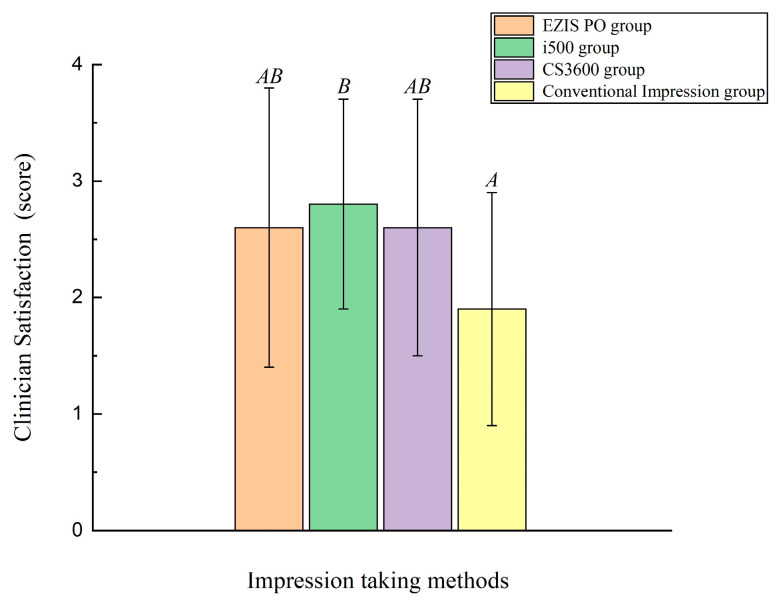
Comparison of clinician satisfaction score of ceramic restorations. Same uppercase letters denote that the difference was not significant (*p* > 0.05).

There were significant differences in the clinician satisfaction score of the ceramic crowns fabricated with three intraoral scanner types and one desktop scanner type (*p* = 0.04; Figure 6; Table 2). The value of the clinician satisfaction score was lower in the desktop scanner as compared to that in the three intraoral scanner types (*p* < 0.001; Figure 6; Table 2), while there were no significant differences as per the three intraoral scanner types (*p* > 0.05; Figure 6; Table 2).

Clinician satisfaction score and marginal fit (absolute marginal discrepancy and marginal gap) had a significant correlation (*p* < 0.05; Table 3). The clinician satisfaction score and absolute marginal discrepancy showed a weak negative correlation (*p* = 0.015; PCC = −0.271; Table 3); the clinician satisfaction score and marginal gap showed a general negative correlation (*p* < 0.001; PCC = −0.403; Table 3).

## 4. Discussion

The present clinical study aimed to fabricate ceramic crowns using the conventional method and the digital method with three different intraoral scanners and compare the marginal and internal fit of the ceramic crowns with clinician satisfaction. Thus, the first null hypothesis stated that there would be no differences between the marginal and internal fit of the ceramic crowns fabricated using the four methods and clinician satisfaction; however, this hypothesis was false for all the ceramic crowns (*p* < 0.001). The second null hypothesis stated that there would be no correlation between the marginal and internal fit of the ceramic crowns and clinician satisfaction; this hypothesis was partially dismissed only with respect to the correlation between the marginal fit and clinician satisfaction (*p* < 0.001).

In Chairside CAD/CAM workflow, the use of an intraoral scanner is essential [11,12]. However, previous studies did not investigate the impact of the type of intraoral scanner in the clinical environment on the marginal and internal fit of the ceramic crown. The present results suggest that conventional impression and intraoral scanner type may affect the marginal and internal fit of the ceramic prostheses and the clinician’s satisfaction score; thus, clinicians should consider the method for acquiring a virtual cast for the fabrication of an excellent ceramic crown.

Most previous studies report the goodness-of-fit of the prostheses based on the type of the restoring material [13,14]. Moreover, these studies mostly evaluated if the marginal fit could be applied in the clinical setting [15,16]. In many previous studies, the clinically allowable range of the marginal fit is assumed to be a value from 100 µm to 120 µm [17,18,19], and a range of 50–100 µm is recommended for the internal fit [20,21,22]. In the present trial, all the ceramic crowns that were fabricated using the conventional method and the digital method with three different intraoral scanners were in the clinically allowable range of marginal fit. However, the internal fit (angle and occlusal gap) had a value exceeding the gap of 100 µm except for CS3600 Group.

Many previous studies have compared the marginal fit and internal fit of the prostheses that was fabricated as per various dental CAD/CAM workflows. Ortorp A. et al. reported poorer marginal fit in the digital workflow (222.5 ± 124.6 µm) as compared to that in the conventional workflow (118 ± 49.7 µm) [23]. Varol S. et al. reported poorer marginal fit in the digital workflow (86.17 ± 27.61 µm) as compared to that in the conventional workflow (77.26 ± 29.23 µm) [24]. In a similar manner, Bayramoglu E. et al. reported poorer marginal fit in the digital workflow (120.4 ± 54.5 µm) as compared to that in the conventional workflow (75.4 ± 16.6 µm) [25]. However, Massignan Berejuk H. et al. reported poorer marginal fit in the conventional workflow (11.56 ± 8.74 µm) as compared to that in the digital workflow (1.85 ± 1.50 µm) [26]. Previous studies have shown different results. Several earlier researches have reported superior marginal fit of prostheses fabricated using the conventional method in a working cast with a physical impression material than that with an intraoral scanner [23,24,25]. However, recently, the use of chairside CAD/CAM workflow has increased, and many intraoral scanners have recently been developed [27,28]. Thus, most recent studies have reported better marginal fit of prostheses fabricated using an intraoral scanner than that of those fabricated using the conventional method [27,28]. In keeping with these results, in the present study, the method for fabrication with three kinds of intraoral scanner showed better marginal and internal fit of the prostheses than the conventional method. As per a systematic review of the multi-unit fixed dental prosthesis fabricated using the digital workflow, Russo LL et al. reported that studies of a single crown fabricated with the digital workflow are generally conducted; however, few studies of a multi-unit fixed dental prosthesis have been performed, and it is important to perform additional studies to confirm the clinical reliability of the findings [29]. Thus, in addition to the evaluation of the single ceramic crown conducted in the present study, it is necessary to perform a study on the multi-unit fixed dental prosthesis.

In the present clinical study, we found a significant correlation between the clinician satisfaction score and the marginal fit (absolute marginal discrepancy and marginal gap) (*p* < 0.05). However, there was no correlation between the clinician satisfaction score and the internal fit (*p* > 0.05) because the marginal fit was recognized as the most important factor in the process wherein clinicians check the prostheses in the oral cavity. Many previous studies have reported that marginal fit is an important element that influences the prognosis of the fixed dental prosthesis [13,20,21,22,23,24,25,26]. Based on the present results, clinicians can use the intraoral scanning method rather than the conventional method for the fabrication of ceramic crowns with excellent goodness-of-fit and realize high clinician satisfaction by reproducing the excellent goodness-of-fit obtained using the intraoral scanning method.

Previous studies have shown a difference in the scanning accuracy based on the intraoral scanner used [11,12]. In the present study, there were significant differences in the marginal fit and internal fit of the ceramic crowns that were fabricated, based on the three intraoral scanner types (*p* < 0.001); however, all values were within the clinically allowable range (within 120 µm), and there were no big differences. Moreover, there were no significant differences in the clinicians’ satisfaction among the ceramic crowns fabricated with the three intraoral scanner types (*p* > 0.05). It is judged that there was no impact on the clinician satisfaction because all the ceramic crowns fabricated with the three intraoral scanner types were in the clinically allowable range (within 120 µm). Further, it is necessary to conduct additional studies to evaluate the three intraoral scanner types used in the present study and examine the impact of the scanning accuracy on the marginal fit and the internal fit.

The present clinical study has certain limitations. It is necessary to conduct additional studies on various intraoral scanners other than those used in the present study. Moreover, it is crucial to perform additional studies using materials other than the lithium disilicate glass-ceramic material that was used in the present clinical study, such as zirconia. We believe that it is important to conduct a study of the multi-unit fixed dental prosthesis. Finally, additional studies on the prognosis should be conducted as a continuation of the present clinical study.

## 5. Conclusions

Based on the findings of this clinical study, the following conclusions were drawn:

1. There was an impact on the marginal and internal fit of the ceramic crowns based on the type of intraoral scanner that was used; however, there was no difference in the clinicians’ satisfaction with the prostheses.
2. The ceramic crowns fabricated using an intraoral scanner showed superior marginal fit and internal fit as well as higher clinician satisfaction than those fabricated using the conventional method with PVS impression.
3. The excellent marginal fit of the fabricated ceramic crowns can achieve high clinician satisfaction.
4. Thus, clinicians should consider the use of the impression method for fabricating a ceramic crown with excellent goodness-of-fit and can realize high clinician satisfaction by reproducing excellent goodness-of-fit using the intraoral scanning method rather than the conventional method.

## Figures and Tables

**Table 1:** Comparison of the discrepancy (µm) of ceramic crowns fabricated with the intraoral scanners and conventional impression technique.

Measurement Position	Discrepancy (Mean ± SD)				F	*p*
	Intraoral Scanner Group			Conventional Impression Technique		
	EZIS PO	i500	CS3600			
**Absolute Marginal Discrepancy**	61.6 ± 11.5 ^A^	67.4 ± 10.2 ^A,B^	71.3 ± 13.5 ^B,C^	78.4 ± 6 ^C^	8.758	<0.001 *
**Marginal Gap**	49.1 ± 8.8 ^A^	49.1 ± 7.7 ^A^	56.5 ± 12.7 ^A^	68.4 ± 8.3 ^B^	17.771	<0.001 *
**Chamfer Gap**	65.9 ± 12.7 ^A^	101.9 ± 6.9 ^B^	78.5 ± 18.8 ^C^	99.5 ± 9.3 ^B^	36.483	<0.001 *
**Axial Gap**	49.2 ± 10.6 ^A^	58 ± 10 ^B^	71.6 ± 8.6 ^C^	71.3 ± 8.3 ^C^	26.547	<0.001 *
**Angle Gap**	102.8 ± 12.9 ^A,B^	122.4 ± 11.2 ^C^	95.7 ± 12.8 ^A^	111.2 ± 9 ^B^	19.505	<0.001 *
**Occlusal Gap**	104.5 ± 11.6 ^A^	133.5 ± 11 ^B^	80 ± 13.9 ^C^	103.7 ± 9.9 ^A^	69.547	<0.001 *

* *p* < 0.05; significance was determined using one-way ANOVA. Different letters (A, B, C) indicate that the difference between the groups was significant, as determined using Tukey’s HSD post hoc test (*p* < 0.05).

**Table 2:** Comparison of the marginal and internal fit of ceramic crowns fabricated with the intraoral scanners and conventional impression technique.

	Intraoral Scanner Group			Conventional Impression Technique	F	*p*
	EZIS PO	i500	CS3600			
**Clinician satisfaction** **(Mean ± SD, Score)**	2.6 ± 1.2 ^A,B^	2.8 ± 0.9 ^B^	2.6 ± 1.1 ^A,B^	1.9 ± 1 ^A^	2.909	0.04 *

* *p* < 0.05; significance was determined using one-way ANOVA. Different letters (A, B, C) indicate that the difference between the groups was significant, as determined by Tukey’s HSD post hoc test (*p* < 0.05).

**Table 3:** Correlation coefficient between the clinical satisfaction score and the marginal and internal fit.

**Clinician Satisfaction**	**Absolute Marginal Discrepancy**		**Marginal Gap**		**Chamfer Gap**	
	***p***	**PCC**	***p***	**PCC**	***p***	**PCC**
	0.015	−0.271	<0.001	−0.403	0.207	-
	**Axial Gap**		**Angle Gap**		**Occlusal Gap**	
	***p***	**PCC**	***p***	**PCC**	***p***	**PCC**
	0.166	-	0.526	-	0.457	-
